# Anti-Insulin Receptor Autoantibodies Are Not Required for Type 2 Diabetes Pathogenesis in NZL/Lt Mice, a New Zealand Obese (NZO)-Derived Mouse Strain

**DOI:** 10.1080/15438600490478029

**Published:** 2004

**Authors:** Marcia F. McInerney, Sonia M. Najjar, Deanna Brickley, Mary Lutzke, George A. Abou-Rjaily, Peter Reifsnyder, Bradford D. Haskell, Kevin Flurkey, Ying-Jian Zhang, Susan L. Pietropaolo, Massimo Pietropaolo, James P. Byers, Edward H. Leiter

**Affiliations:** 1 Departments of Medicinal and Biological Chemistry and Pharmacology College of Pharmacy University of Toledo 2801 W. Bancroft Street, BO 2833, Mail Stop 606 Toledo OH 43606 USA; 2 Department of Pharmacology and Therapeutics Medical College of Ohio Toledo Ohio USA; 3 The Jackson Laboratory Bar Harbor Maine USA; 4 University of Pittsburgh School of Medicine Diabetes Institute Pittsburgh Pennsylvania USA

## Abstract

The New Zealand obese (NZO) mouse strain shares with the related New Zealand black (NZB) strain a number of immunophenotypic traits. Among these is a high proportion of B-1 B lymphocytes, a subset associated with autoantibody production. Approximately 50% of NZO/HlLt males develop a chronic insulin-resistant type 2 diabetes syndrome associated with 2 unusual features: the presence of B lymphocyte–enriched peri-insular infiltrates and the development of anti-insulin receptor autoantibodies (AIRAs). To establish the potential pathogenic contributions ofBlymphocytes and AIRAs in this model, a disrupted immunoglobulin heavy chain gene (*Igh-6*) congenic on the NZB/BlJ background was backcrossed 4 generations into the NZO/HlLt background and was then intercrossed to produce mice that initially segregated for wild-type versus the mutant *Igh-6* allele and thus permitted comparison of syndrome development. A new flow cytometric assay (AIRA binding to transfected Chinese hamster ovary cells stably expressing mouse insulin receptor) showed IgM and IgG subclass AIRAs in serum from *Igh-6* intact males, but not in Igh6^null^ male serum. However, the absence of B lymphocytes and antibodies distinguishing mutant from wild-type males failed to significantly affect diabetes-free survival. The Igh6^null^males gained weight less rapidly than wild-type males, probably accounting for a retardation, but not prevention, of hyperglycemia. Thus, AIRA and the Blymphocyte component of the peri-insulitis in chronic diabetics were not essential either to development of insulin resistance or to eventual pancreatic beta cell failure and loss. A new substrain, designated NZL, was generated by inbreeding *Igh-6* wild-type segregants. Currently at the F10 generation, NZL mice exhibit the same juvenile-onset obesity as NZO/HlLt males, but develop type 2 diabetes at a higher frequency (> 80%). Also, unlike NZO/HlLt mice that are difficult to breed, the NZL/Lt strain breeds well and thus offers clear advantages to obesity/diabetes researchers.


*Experimental Diab. Res.*, 5:177–185, 2004
Copyright © Taylor and Francis Inc.
ISSN: 1543-8600 print / 1543-8619 online
DOI: 10.1080/15438600490478029

### Anti-Insulin Receptor Autoantibodies Are Not Required for Type 2 Diabetes Pathogenesis in NZL/Lt Mice, a New Zealand Obese (NZO)-Derived Mouse Strain

**Marcia F. McInerney,[1] Sonia M. Najjar,[2] Deanna Brickley,[1] Mary Lutzke,[1] George A. Abou-Rjaily,[2] Peter Reifsnyder,[3] Bradford D. Haskell,[3] Kevin Flurkey,[3] Ying-Jian Zhang,[4] Susan L. Pietropaolo,[4] Massimo Pietropaolo,[4] James P. Byers,[1] and Edward H. Leiter[3]**

[1]*Departments of Medicinal and Biological Chemistry and Pharmacology, University of Toledo College of Pharmacy, Toledo, Ohio, USA*
[2]*Department of Pharmacology and Therapeutics, Medical College of Ohio, Toledo, Ohio, USA*
[3]*The Jackson Laboratory, Bar Harbor, Maine, USA*
[4] *University of Pittsburgh School of Medicine Diabetes Institute, Pittsburgh, Pennsylvania, USA*

**The New Zealand obese (NZO) mouse strain shares with the related New Zealand black (NZB) strain a number of immunophenotypic traits. Among these is a high proportion of B-1 B lymphocytes, a subset associated with autoantibody production. Approximately 50% of NZO/HlLt males develop a chronic insulin-resistant type 2 diabetes syndrome associated with 2 unusual features: the presence of B lymphocyte–enriched peri-insular infiltrates and the development of anti-insulin receptor autoantibodies (AIRAs). To establish the potential pathogenic contributions of B lymphocytes and AIRAs in this model, a disrupted immunoglobulin heavy chain gene (*Igh-6*) congenic on the NZB/BlJ background was backcrossed 4 generations into the NZO/HlLt background and was then intercrossed to produce mice that initially segregated for wild-type versus the mutant *Igh-6* allele and thus permitted comparison of syndrome development. A new flow cytometric assay (AIRA binding to transfected Chinese hamster ovary cells stably expressing mouse insulin receptor) showed IgM and IgG subclass AIRAs in serum from *Igh-6* intact males, but not in *Igh-6*$^{null}$ male serum. However, the absence of B lymphocytes and antibodies distinguishing mutant from wild-type males failed to significantly affect diabetes-free survival. The *Igh-6*$^{null}$ males gained weight less rapidly than wild-type males, probably accounting for a retardation, but not prevention, of hyperglycemia. Thus, AIRA and the B-lymphocyte component of the peri-insulitis in chronic diabetics were not essential either to development of insulin resistance or to eventual pancreatic beta cell failure and loss. A new substrain, designated NZL, was generated by inbreeding *Igh-6* wild-type segregants. Currently at the F10 generation, NZL mice exhibit the same juvenile-onset obesity as NZO/HlLt males, but develop type 2 diabetes at a higher frequency (>80%). Also, unlike NZO/HlLt mice that are difficult to breed, the NZL/Lt strain breeds well and thus offers clear advantages to obesity/diabetes researchers.**

**Keywords** Autoantibodies; B Lymphocytes; Insulin Receptor; New Zealand Obese (NZO) Mice; Type 2 Diabetes Mellitus

Received 30 March 2004; accepted 24 April 2004.

The McInerney laboratory was supported by the Diabetes Action Research and Education Foundation (grant nos. 105 and 126). This work was partially supported by NIH research grants DK 54254 and DK 57497 and a research grant from the American Diabetes Association to SMN, and by NIH research grant DK 56853 to EHL. Institutional shared services were supported by National Cancer Institute Cancer Support Grant CA-34196 to The Jackson Laboratory. This work was also supported by NIH grants R01 DK53456 and R01 DK56200 (M. Pietropaolo). The authors acknowledge Drs. Hermann von Grafenstein and Katherine Wall for critical reading of the manuscript.

Address correspondence to Marcia F. McInerney, PhD, Department of Medicinal and Biological Chemistry, College of Pharmacy, University of Toledo, 2801 W. Bancroft Street, BO 2833, Mail Stop 606, Toledo, OH 43606, USA. E-mail: mmciner@utnet.utoledo.edu

New Zealand obese (NZO) is an inbred mouse strain derived in New Zealand from outbred stock from the Imperial Cancer Research Fund Laboratories in London [1]. NZO mice have been studied primarily as a mouse model of obesity-induced insulin-resistant diabetes [2]. Males of the NZO/HlLt strain develop diabetes at a frequency of 40% to 50% whereas NZO/HlLt females develop marked obesity without diabetes [3]. Because of the early development of obesity, the strain is difficult to breed and has not been widely studied. Thus, despite the relatedness of the NZO strain to the autoimmune-prone New Zealand black (NZB) and New Zealand white (NZW) strains, only limited analysis of the immune system of NZO mice has been reported [4, 5]. These studies indicated that NZO shared some of the classic autoimmune abnormalities found in the NZB strain, and suggested that the development of diabetes is associated with autoimmunity. Among the immune anomalies reported were the development of immunoglobulin M (IgM) autoantibodies to native and single-stranded DNA as well as IgM immune complex deposition in kidney [4]. Another immune anomaly shared with the NZB strain was splenic hypertrophy with increased basal unstimulated splenocyte proliferation in vitro, but reduced mitogen-stimulated proliferation. However, this hypoproliferative phenotype was reversed by insulin administration only in NZO, but not in NZB mice [5], suggesting that the immune anomaly in NZO was related to insulin resistance in these mice. That autoimmunity may contribute, in part, to the metabolic syndrome in NZO mice was suggested by the report that this strain spontaneously develops autoantibodies to the insulin receptor [6]. Recent work from our laboratory found a high frequency of B1 B lymphocytes in NZO/HlLt mice, a subset associated with autoantibody production [7]. Furthermore, pancreatic histopathology of the peri-insular infiltrates commonly observed in chronically diabetic NZO/HlLt males showed a higher frequency of B lymphocytes than T lymphocytes and included plasma cells (antibody producers) [8]. To establish whether the type 2 diabetes syndrome developing in NZO/HlLt males was dependent upon humoral immunity, we developed a new flow cytometry–based methodology for detecting anti-insulin receptor autoantibody (AIRA) and generated a B lymphocyte–deficient stock on the NZO strain background for comparison of syndrome development in the presence versus absence of AIRAs and other autoantibodies.

### METHODS

### Mice

NZO/HlLt and C57BL/6J mice were housed in a specific pathogen–free (SPF) environment at The Jackson Laboratory. NZO/HlLt male progeny were aged to 24 weeks, with body weight measured every 2 weeks and plasma glucose (glucose analyzer; Beckman Instruments, Palo Alto, CA) measured every 4 weeks. Diabetes (plasma glucose levels >250 mg/dL) was 50% in NZO males by 24 weeks of age.

All animals were handled in accordance with the guidelines of the National Institutes of Health, and the Institutional Animal Care and Use Committee of The Jackson Laboratory.

### Generation of a New Recombinant Congenic Strain (NZL/Lt) Segregating for B-Lymphocyte Deficiency

NZB/BlnJ mice congenic for a disrupted *Igh-6* allele on chromosome 12 encoding the IgM heavy chain [9] (formal designation NZB,129-*Igh-6*$^{\mathrm{tm1Cgn}}$) were kindly provided by Dr. L. D. Shultz (The Jackson Laboratory) [10]. Males from this stock (at N11) were outcrossed with NZO/HlLt females and F1 females were backcrossed to NZO/HlLt males. In 4 subsequent backcross cycles, heterozygous *Igh-6*$^{null}$ carriers were identified by polymerase chain reaction (PCR) for the neomycin resistance cassette used in gene targeting. At N5, heterozygotes were intercrossed, and N5F1 *Igh-6*$^{null}$ homozygotes were identified by flow cytometric demonstration of the absence of B220$^+$ B lymphocytes in peripheral blood (monoclonal antibody [mAb] clone RA3-6B2; BD PharMingen). Homozygous mice generated between N5F1 and N5F4 were analyzed. At N5F2, a control line, now designated NZL, was selected that was wild type (e.g., NZO alleles) at the *Igh-6* locus and at flanking polymorphic microsatellite markers distinguishing NZO from NZB.

### Analysis of Diabetic Syndrome Progression

Wild-type NZL and NZL-*Igh-6*$^{null}$ males were accumulated between N5F1 and N5F4 and longitudinally profiled for biweekly changes in body weight (BW) and nonfasting plasma glucose (PG) determined on a glucose analyzer (Beckman Instruments, Fullerton, CA). Mice were maintained on a 6% fat-containing chow (NIH-31; Purina, Richmond, IN) and acidified drinking water. Because the immunodeficient NZL-*Igh-6*$^{null}$ mice were very susceptible to infections, both genotypes were maintained in pressurized individually ventilated (PIV) caging and received sulfamethoxazole thiomethoprim (Goldline Laboratories, Ft. Lauderdale, FL) for 3 days per week. Serum samples for AIRA analysis from both genotypes were collected at the 20-week termination point for analysis in the AIRA assay (see below).

### Generation of Chinese Hamster Ovary Mouse Insulin Receptor–Transfected Cell Line

Full length ($\alpha$ and $\beta$ chain) mouse insulin receptor (mIR) cDNA was excised from pMTIII (M. Daniel Lane; Johns Hopkins, Baltimore, MD) [11] by *NotI*/*SalI* restriction digestion and subcloned into pREP4-hygromycin–resistant mammalian expression vector at Not1/Xho1 sites (Invitrogen, Carlsbad, CA).

Chinese hamster ovary (CHO) cells (American Type Culture Collection, Rockville, MD) were transfected with 10 $\mu$g of the pREP4/mIR construct using LipofectAMINE PLUS (Gibco BRL, Gaithersburg, MD) per manufacturer's instructions. Transfected CHO cells were grown in F12K media (Gibco) with 10% fetal calf serum and selected with 600 $\mu$g/mL hygromycin B (Calbiochem, LaJolla, CA). Hygromycin-resistant clones were screened for IR expression by $^{125}$I-insulin–binding assay [12, 13]. HIR3.5 (Jonathan Whittaker; NYU School of Medicine, NY, NY), stably transfected NIH 3T3 fibroblasts expressing $10^6$ human IRs per cell [14], served as a positive control in the screening experiments. Untransfected parental CHO cells were used as a negative control to account for any background expression of endogenous hamster IRs. Clone 36, the transfected CHO clone with the highest level of mouse IR expression, was labeled with fluorescein isothiocyanate (FITC)-labeled insulin (Sigma, St. Louis, MO) and cells expressing high density of IRs were positively sorted using a Becton-Dickinson flow cytometer (Wayne State University, Detroit, MI). Sorted cells were subjected twice to limiting dilution until a subclone (mIR36.11.1) with stable cell surface expression of mIR was obtained. Subclone mIR36.11.1, untransfected parental CHO cells, and positive control HIR3.5 were retested for IR expression by $^{125}$I-insulin–binding assay [12, 13].

### Functionality of the mIR on the mIR 36.11.1 CHO Cells–Transfected Clone

Specific binding of $^{125}$I-insulin (human recombinant; Amersham Biosciences, Piscataway, NJ) on mIR36.11.1 and control HIR 3.5 cells was defined by competition with unlabeled insulin as previously described [12, 13]. Briefly, $^{125}$I-insulin (50,000 cpm, $11.36 \times 10^{-12}$ M) was added to cells ($1 \times 10^6$ per well) either alone to determine maximum binding or in the presence of increasing concentrations of unlabeled insulin ($10^{-10}$ to $10^{-6}$ M) in a final incubation volume of 1 mL. Following binding overnight at 4°C, cells were washed with phosphate-buffered saline (PBS; JRH Biosciences, Lenexa, MO), and the amount of bound $^{125}$I-insulin in 0.4 N NaOH–treated cells was counted in a Beckman Gamma 5000 counter (Medical College of Ohio, Toledo, OH). All samples were done in duplicate.

Specific binding of biotinylated insulin was also defined by competition with unlabeled insulin using the fluorescence activated cell sorter (FACS) analysis. mIR36.11.1 cells at $5 \times 10^5$ were incubated with $5 \times 10^{-6}$ M biotinylated insulin (a gift from Dr. Francis Finn, University of Pittsburg, retired) either alone to determine maximum binding or in the presence of increasing concentrations of unlabeled insulin ($10^{-9}$ to $10^{-3}$ M) for 45 minutes on ice followed by washing. The secondary reagent streptavidin-phycoerythrin (SA-PE; Pharmingen, San Diego, CA) was then added for 45 minutes on ice. Control cells were treated with 30 $\mu$g/mL unlabeled insulin and SA-PE following the procedure above to determine background binding. After washing, cells (10,000 per sample) were analyzed immediately using a Becton-Dickinson flow cytometer (University of Toledo, Toledo, Ohio) to determine the geometric mean fluorescence (GMF) of each sample.

### Anti-mIR Antibody Assay Using mIR-Transfected CHO Cells and Flow Cytometry

Serum AIRA was quantified by FACS analysis of mouse Ig binding to mIR 36.11.1 compared to untransfected CHO cells. NZO/HlLt sera used in flow cytometry were precipitated by 25% saturated ammonium sulfate (SAS) to obtain an IgM-enriched fraction. The supernatant was then subjected to an additional 45% SAS precipitation and purified further by protein A conjugated beads to enrich for IgG antibodies. Sample binding to untransfected CHO cells was used to establish a baseline in FACS analysis. Incubation with biotinylated insulin and SA-PE served as a positive control for staining. Serum samples were pooled from 5 to 6 mice from each of the following: C57BL/6J female 30 weeks of age, C57BL/6J males 30 weeks of age, NZO/HlLt female 16 to 17 weeks of age, NZO/HlLt females 34 weeks of age, NZO/HlLt males 16 to 17 weeks of age, and NZO/HlLt males 20 to 28 weeks of age, all with normal blood glucose levels. An additional group of 9 diabetic males between 20 and 28 weeks of age (plasma glucose >250 mg/dL) were also analyzed. In a separate set of experiments serum was collected from 20-week-old male NZL (pool of 5), NZL-*Igh-6$^{null}$* (pool of 10), and NZO diabetic mice (pool of 5). IgM and IgG fractionation on pooled serum samples was performed using a protein G spin chromatography kit (Pierce, Rockford, IL) according to manufacturer's instructions. Fractionated antibody (Ab) was added to $0.5 \times 10^6$ cells for 45 minutes on ice, washed off and replaced by secondary antibody, affinity purified F(ab')$_2$ donkey anti-mouse IgG (H + L)-PE (minimal cross-reactivity with bovine, chicken, goat, guinea pig, hamster, horse, human, rabbit, or sheep serum proteins; Jackson Immunologicals, West Grove, PA). After an incubation of 45 minutes on ice, cells were washed, iced, and analyzed immediately using a Coulter Elite flow cytometer (Medical College of Ohio, Toledo, OH) or a Becton-Dickinson flow cytometer (University of Toledo, Toledo, OH). No detectable shift was seen with parental or transfected mIR 36.11.1 cells labeled with secondary antibody alone or SA-PE alone.

### Radioimmunoassay with the $\beta$ Subunit of the Human IR

AIRAs that were detected by flow cytometry analysis of Ig-binding to mIR were also detected by radioimmunoassay. Sera from NZO/HlLt and control mice were used to precipitate the in vitro transcribed/translated $^{35}$S-Met–labeled $\beta$ subunit of the human IR. Protein G was applied to precipitate IgG immunocomplexes and several washes were preformed to remove any unbound antigen. Results are expressed in cpm. A rabbit anti-human IR $\beta$ subunit polyclonal antisera was used as positive control (Research Diagnostic, Flanders, NJ) and normal rabbit serum as negative control.

### Statistics

Survival statistics were performed using Kaplan-Meier analysis (STATVIEW; Abacus Software, Palo Alto, CA). Significance of differences for other data were analyzed using ANOVA with Bonferroni/Dunn correction or the Student's $t$ test with significant differences set at a $P$ value of less than .05.

### RESULTS

### Functionality of the mIR on the mIR36.11.1-Transfected CHO Clone

In order to develop an assay for the detection of antibodies to the IR, we transfected CHO cells with an IR-coding plasmid. IR-expressing cells were screened by $^{125}$I-insulin–binding assay [12, 13], sorted by flow cytometry using FITC-insulin, cloned, and subcloned by limiting dilution until a stable IR-expressing CHO transfectant clone was isolated. Positive control HIR3.5 and mIR-transfected CHO subclone 36.11.1 (mIR36.11.1) cells bound $^{125}$I-insulin to a comparable extent and significantly more effectively than untransfected CHO cells ($P < .03$) (Figure 1*A*).

The specific binding of $^{125}$I-insulin to the IR on HIR3.5 and mIR36.11.1 cells was analyzed by competition with increasing concentrations of unlabeled insulin ($10^{-10}$ to $10^{-6}$ M). As shown in Figure 1*B* (HIR3.5) and Figure 1*C* (mIR36.11.1), increasing concentrations of unlabeled insulin competed for the insulin binding site and prevented binding of a constant concentration of $^{125}$I-insulin. $^{125}$I-insulin binding was completely inhibited with $2 \times 10^{-6}$ M unlabeled insulin in both cell lines. Furthermore, based on Scatchard analysis, the HIR3.5 cell line expressed $0.5 \times 10^{6}$ IRs per cell as compared to the mIR36.11.1 clone that expressed $2 \times 10^{6}$ IRs per cell.

In comparison to the flow cytometric profile of untransfected CHO cells, a rightward shift of fluorescence intensity was observed following labeling of the stable mIR36.11.1 transfectant subclone with biotinylated insulin and SA-PE, confirming cell surface expression of mIR (Figure 1*D*). Specific binding of biotinylated insulin was confirmed by competitive binding in the presence of increasing concentrations of unlabeled insulin ($10^{-9}$ to $10^{-3}$ M). Unlabeled insulin competed with biotinylated insulin for the insulin binding site on mIR36.11.1 cells (Figure 1*E*). Binding of biotinylated insulin was completely inhibited in the presence of $5 \times 10^{-4}$ M unlabeled insulin (Figure 1*E*). Overall, the results from Figure 1 show that mIR36.11.1 cells express IR (Figure 1*A* and *D*) comparable to that expressed in the positive-control HIR3.5 cells (Figure 1*A* and Scatchard results) and insulin specifically binds the IR expressed in mIR36.11.1 cells (Figure 1*C* and *E*). Furthermore, the transfected IR in mIR36.11.1 was tyrosine phosphorylated in the presence of insulin (data viewable at the website http://mbc.pharm.utoledo.edu/mbc/mfm.html), indicating that IR in mIR36.11.1 cells is functionally capable of insulin signal transduction.

### AIRAs in NZO Mice

The use of the IR-transfected CHO cell system permitted confirmation of the previous report of low-affinity IgM AIRAs present in NZO serum [6]. Figure 2*A* (extreme right hand bars) documents that the enriched IgM fraction (IgM concentration 9.7 $\mu$g/mL) from NZO/HlLt males at 16 to 17 weeks of age contained AIRAs.

Most pathological autoimmune antibodies undergo a class switch from IgM to IgG isotypes. Therefore, supernatants from 25% SAS cuts were precipitated with 45% SAS and this fraction was passed over protein A columns to obtain samples enriched in IgG. The major protein peak of the acid eluate was collected in a single fraction for all samples except the sample from diabetic NZO/HlLt males, which was sufficiently large to collect in multiple fractions. Specific AIRA was detected in the diabetic 20- to 28-week NZO/HlLt male pool only in the leading fraction from the column that had a relatively low Ig concentration (e.g., IgM 1.3 $\mu$g/mL, IgG1 14 $\mu$g/mL) (Figure 2*A*). However, IgG AIRA was not present in age-matched (20- to 28-week) normoglycemic NZO/HlLt male mice that had more than double the amount of IgG1 (36 $\mu$g/mL). Nor was IgG AIRA detected in any other tested pools when compared to background B6 mice (Figure 2*A*). When immunoprecipitate fractions (IgG enriched) were tested in a radioimmunoassay with $^{35}$S-Met–labeled recombinant $\beta$ chain of the human IR, only the NZO/HlLt serum obtained from diabetic males contained significant amounts of AIRAs (Figure 2*B*). (Diabetic 20- to 28-week NZO/HlLt male versus normal 20- to 28-week NZO/HlLt male, $P < .0005$; diabetic 20- to 28-week versus 30-week C57BL/6J males, $P < .0009$). Therefore, the same results on IgG-enriched AIRAs in diabetic NZO/HlLt male mice were obtained in 2 independent assay systems.

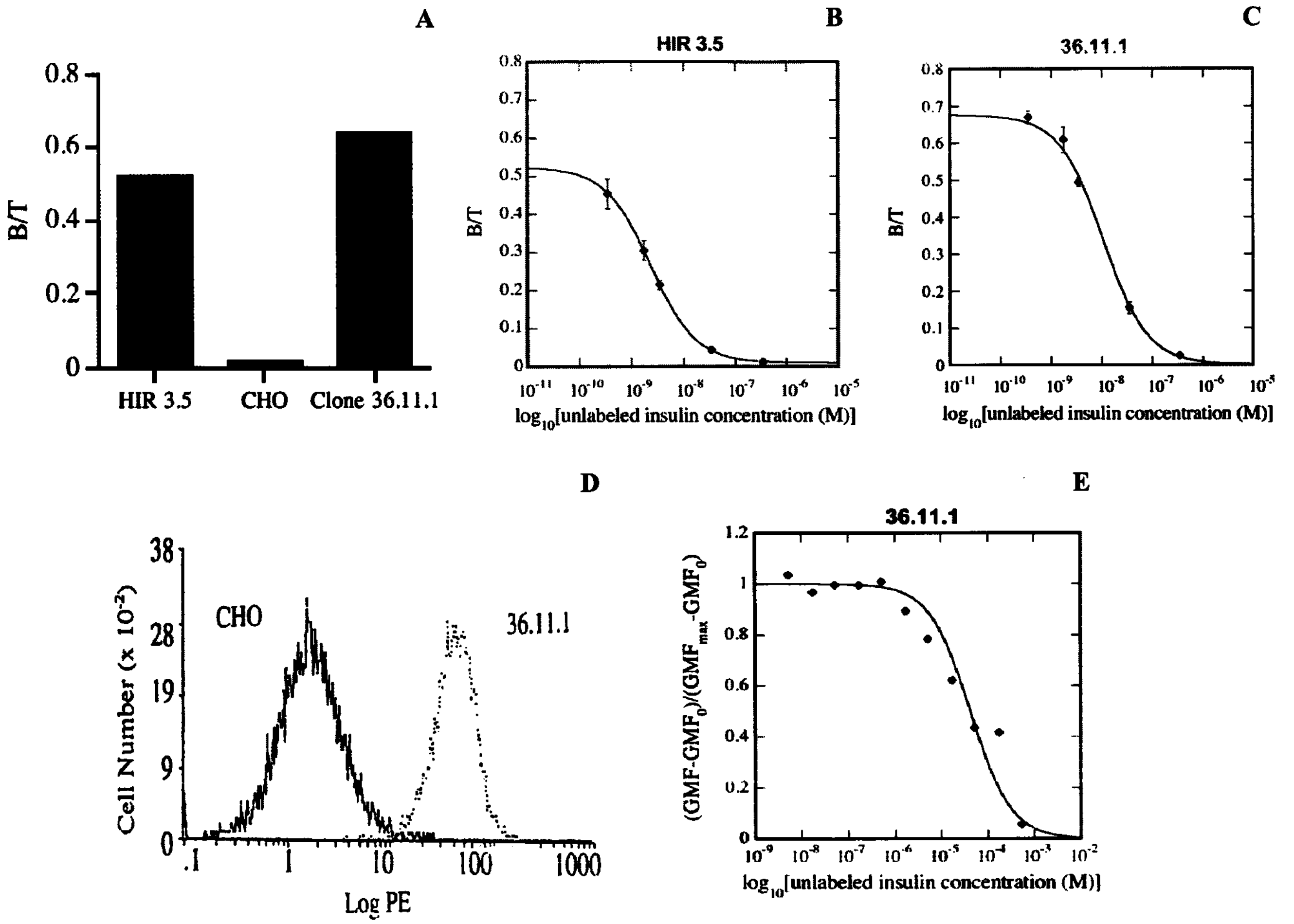

FIGURE 1

(*A*) CHO untransfected parental cells, mouse IR–transfected CHO subclone 36.11.1 (mIR36.11.1), and positive control, human IR–transfected HIR3.5 cells (x-axis) were analyzed for IR expression by $^{125}$I-insulin binding assay [12, 13]. 'Total $^{125}$I-insulin binding' was determined by the following calculation: (counts per minute $^{125}$I-insulin bound per $10^6$ cells per well/50,000 cpm $^{125}$I-insulin added per well) and is presented by bound $^{125}$I-insulin/total $^{125}$I-insulin (B/T) on the y-axis. Duplicate wells of each cell type were analyzed and the standard deviation was no greater than 15%. (*B* and *C*) Duplicate wells of HIR3.5 cells (*B*, $1 \times 10^6$ per well) and mIR36.11.1 cells (*C*, $1 \times 10^6$ per well) were tested for specific binding of $^{125}$I-insulin by competition with increasing concentrations of unlabeled insulin ($10^{-10}$ to $10^{-6}$ M). (*D*) CHO parental cells (*solid line*) and mIR36.11.1-transfected cells (*dotted line*) were labeled with biotinylated insulin and strepavadin-phycoerythrin (SA-PE) and analyzed by flow cytometry. (*E*) mIR36.11.1–transfected cells at $5 \times 10^5$ were labeled with biotinylated insulin either alone or in the presence of increasing concentrations of unlabeled insulin ($10^{-9}$ to $10^{-3}$ M), followed by secondary reagent SA-PE. The y-axis is the [(geometric mean fluorescence (GMF) − $GMF_0$ (background GMF of unlabeled insulin and SA-PE))/($GMF_{max}$ (maximum binding of biotinylated insulin and SA-PE) − $GMF_0$)] as determined by flow cytometry analysis. This graph is representative of 2 experiments.

Furthermore, at least some NZO/HlLt AIRAs are cross reactive, because AIRAs from diabetic NZO/HlLt males bound to the $\beta$ chain of the human IR.

### B Lymphocyte–Deficient NZL-*Igh-6$^{null}$* Mice Do Not Express AIRAs

In order to determine if AIRAs are pathogenic, NZB/BlnJ mice congenic for a disrupted *Igh-6* allele on chromosome 12 encoding the IgM heavy chain [9] were crossed with NZO/HlLt mice as described above to obtain NZL-*Igh-6$^{null}$* mutant and NZL wild-type that express NZO *Igh-6* alleles. Analysis of serum IgM and IgG fractions obtained from NZL wild-type, NZL-*Igh-6$^{null}$*, and NZO males is shown in Figure 3*A*. As expected, B cell–deficient NZL-*Igh-6$^{null}$* mice did not express either IgM or IgG AIRAs whereas wild-type NZL and NZO mice both expressed IgM, and to a lesser extent IgG, AIRAs (Figure 3*A*).

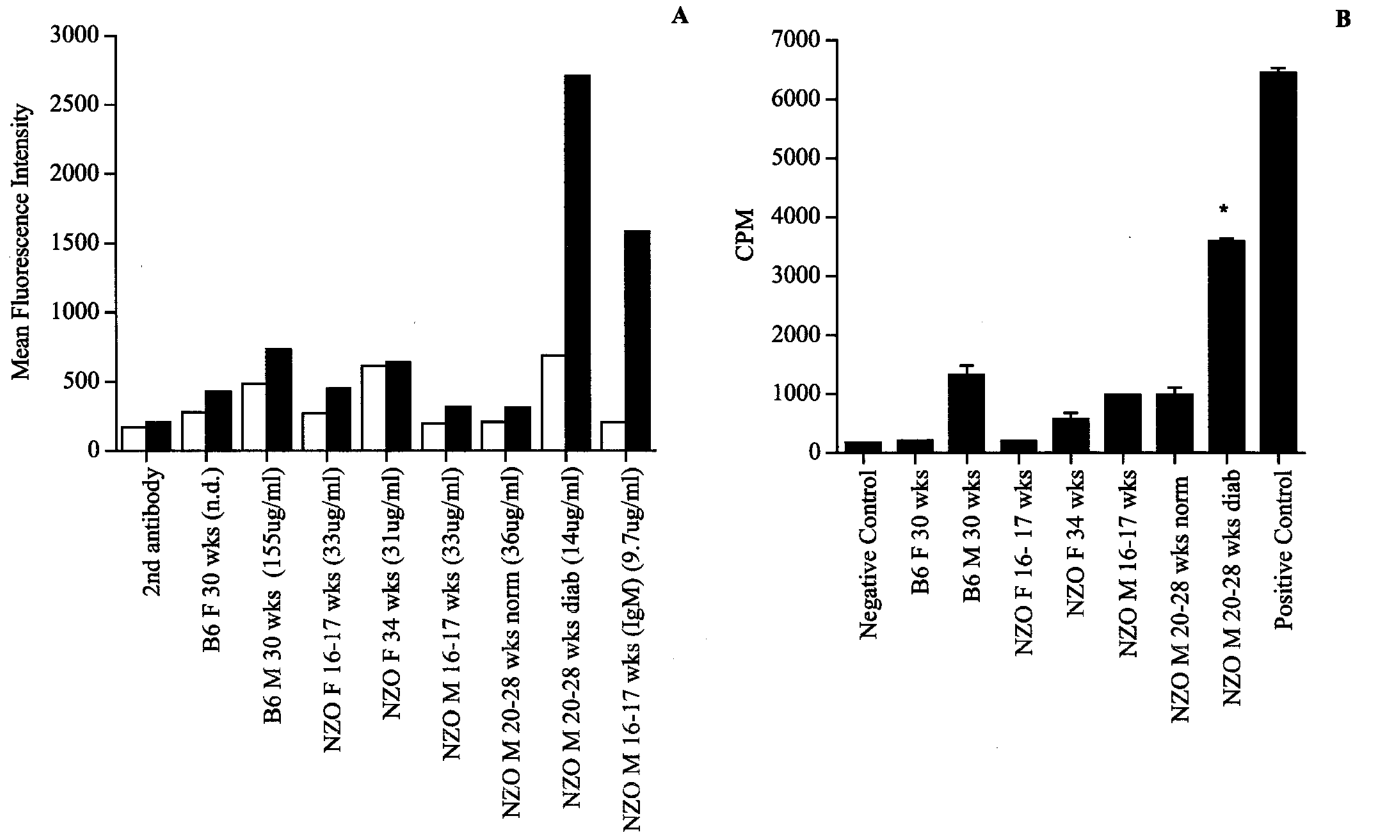

**FIGURE 2**

(*A*) CHO parental cells (*open bars*) and mIR36.11.1 (*solid bars*) were labeled with either 2nd antibody alone [F(ab')$_2$ donkey anti-mouse IgG (H + L)-PE] or an IgG-enriched serum fraction (45% SAS cut of 25% SAS supernatant passed over protein A column) and 2nd antibody. IgG-enriched serum fractions were obtained from pools of mice (5 to 6 mice): 30-week-old female or male C57BL/6J mice (B6 F 30 wks, B6 M 30 wks); 16- to 17-week-old female or male NZO mice (NZO F 16-17 wks, NZO M 16-17 wks); 34-week-old female NZO mice (NZO F 34 wks); and 20- to 28-week-old NZO male mice either normoglycemic or diabetic (9 mice in the diabetic pool) (NZO M 20-28 wks norm, NZO M 20-28 wks diab). IgG1 concentrations are given in parentheses on the graph label on the x-axis for each group tested as an indicator of relative immunoglobulin concentrations. A 25% SAS IgM–enriched fraction, from the 16- to 17-week-old male NZO pool, was also tested and is labeled on the x-axis of the graph as NZO M 16-17 wks (IgM). The IgM concentration of this 25% SAS fraction from 16- to 17-week-old NZO males was 9.7 $\mu$g/mL (see parentheses). The y-axis is the mean fluorescence intensity. The IgG results are representative of duplicate samples and the IgM results are representative of 2 experiments. (*B*) Immunoprecipitation of $^{35}$S-Met–labeled $\beta$ subunit of the human IR by serum fractions from control female and male C57BL/6J mice and female and male NZO nondiabetic and diabetic mice of various ages as described in *A* above. Protein G was applied to precipitated IgG-enriched immunocomplexes. Results are expressed as cpm (mean $\pm$ SD) of at least 2 independent sets of experiments performed in triplicate. *Significance ($P < .0009$) for the difference between diabetic male NZO mice and other strains of mice (C57BL/6J) as well as nondiabetic male and female NZO mice (Student's $t$ test).

### B-Lymphocyte Deficiency Retards, But Does Not Prevent, Diabetes in NZL Males

The genome of the NZL strain (selected for the absence of the targeted *Igh-6* gene and closely linked alleles deriving from 129S2/SvPas on chromosome 12) should derive approximately 97% of its genome from NZO and 3% from the NZB donor strain. The NZL genome was typed at F9 for 38 informative MIT microsatellite markers (Research Genetics, Huntsville, AL) and 116 informative single nucleotide polymorphisms (SNPs; KBioscience, Oxford, England). Although analytic gaps remain on some chromosomes, most of the genome scanned is NZO derived. NZB-derived genome was found only on Chromosome 9 (Mb 34 to 103) and Chromosome 18 (Mb 55 to 80). A map is viewable at http://www.jax.org/staff/leiter/labsite/type2.html. Consistent with the genome scan data, development of severe obesity in

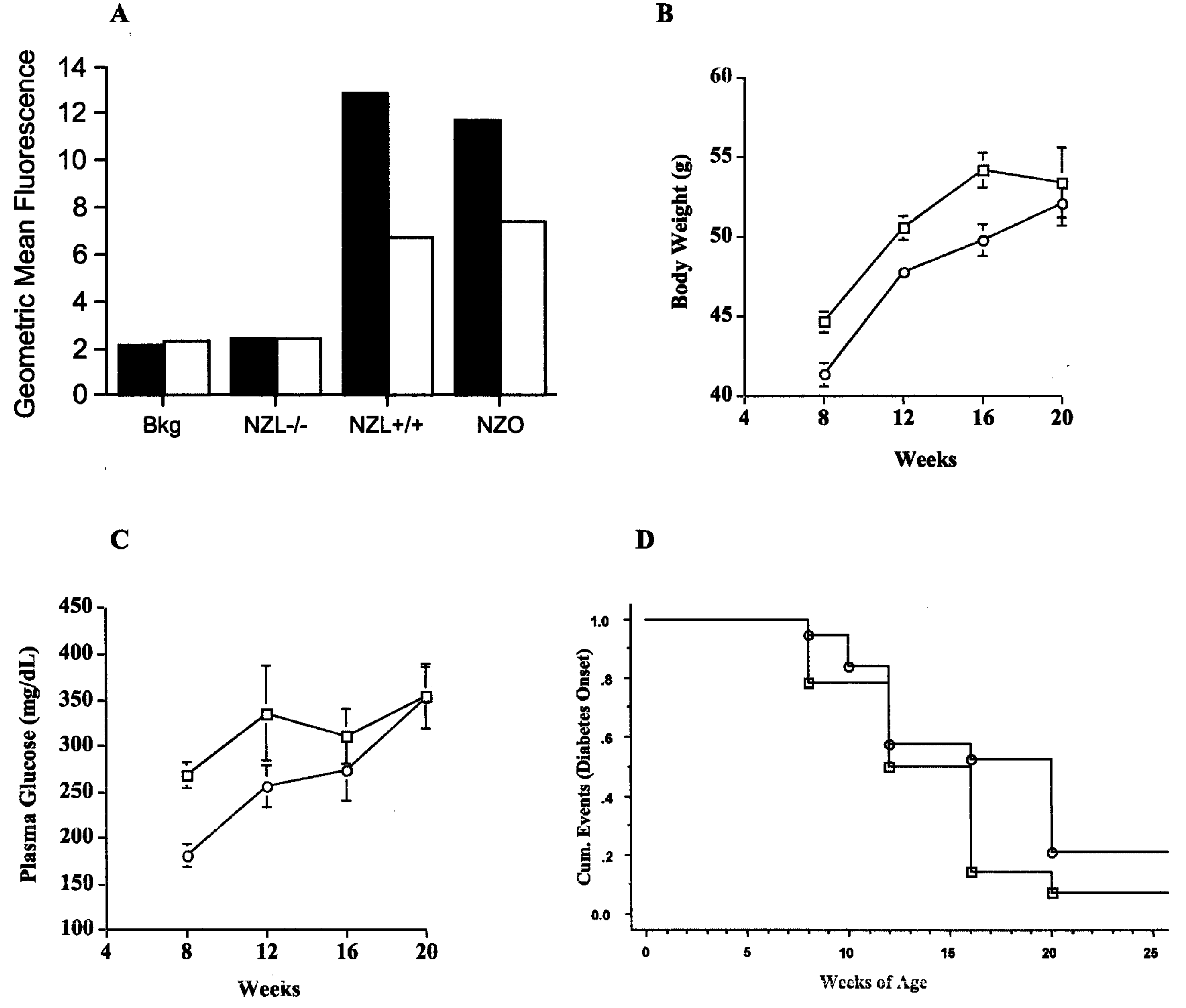

FIGURE 3

(*A*) Enriched IgM and IgG serum fractions were obtained from 20-week-old male NZL wild type (designated as NZL+/+, pool of 5), NZL-*Igh-6*$^{null}$ (designated as NZL−/−, pool of 10), and diabetic NZO mice (pool of 5). mIR 36.11.1 cells were labeled with either 2nd antibody alone [ F(ab')$_2$ donkey anti-mouse IgG (H + L)-PE] or an IgM- (*solid bars*) or IgG- (*open bars*) enriched serum fraction (protein G spin chromatography kit) and 2nd antibody and analyzed by flow cytometry. The y-axis is the mean fluorescence intensity. (*B* to *D*) NZL wild-type males are represented by the open square whereas NZL-*Igh-6*$^{null}$ males are represented by the open circles. (*B*) Comparative changes in body weight in grams (g, mean BW ± SEM) of NZL and NZL-*Igh-6*$^{null}$ males. (*C*) Comparative changes in plasma glucose (mean PG ± SEM) in NZL and NZL-*Igh-6*$^{null}$ males. Values above 250 mg/dL are considered diabetic. (*D*) Onset of diabetes is shown as percent survival regression analysis. There is no significant difference between the 2 genotypes for diabetes incidence. n = 14 for NZL and n = 19 for NZL-*Igh-6*$^{null}$ males.

NZL males (and females, data not shown) was typical of that observed in NZO/HlLt males. NZL-*Igh-6*$^{null}$ males, although markedly obese as well, weighed significantly less between 8 and 16 weeks of age ($P < .01$) (Figure 3*B*). Both NZL wild-type and *Igh-6*$^{null}$ mutant males developed maturity-onset hyperglycemia (Figure 3*C*). At the 8-week sampling, the NZL wild-type mean was significantly higher than the mutant mean; however, the means did not differ at the later time points. Of the groups shown in Figure 3*C*, 13 of 14 wild-type males (93%) and 15 of 19 *Igh-6*$^{null}$ males (79%) exhibited hyperglycemia

>250 mg/dL at the 20-week sampling point. These frequencies are higher than the 50% frequency of diabetes observed in standard NZO/HlLt males [15]. Diabetes-free survival analysis showed no significant differences between the 2 genotypes (Figure 3*D*). Hence, elimination of humoral autoimmunity slightly retarded but did not prevent development of hyperglycemia. Although NZL wild-type mice of both sexes did not differ from standard NZO/HlLt mice in terms of rapid development of juvenile obesity and maturity-onset development of hyperglycemia, a major difference was observed in reproductive performance. Whereas 40% or fewer of NZO matings were productive, almost all NZL pair matings established at weaning were productive. Thus, the recombinant congenic strain exhibits reproductive vigor that makes this strain much easier to breed.

The results in Figure 3 show that the absence of B lymphocytes and correspondingly AIRAs did not protect NZL-*Igh-6$^{null}$* mice from the development of diabetes. Therefore, AIRAs are not essential for the pathogenicity associated with insulin resistance and hyperglycemia in type 2 diabetes in the NZL mouse.

### DISCUSSION

Previously published preliminary results showed that a small cohort of B lymphocyte–deficient NZL-*Igh-6$^{null}$* males did not transit to overt diabetes [7]. However, 2 factors indicated caution in interpretation of this finding. First, diabetes frequency in standard NZO/HlLt males is only 50%. Therefore, the small sample size of B lymphocyte–deficient males (n = 4) available for anlaysis was inconclusive. Second, these immunodeficient males were highly susceptible to infections, with all 4 dying of undiagnosed illness before 20 weeks of age. It has been our further experience maintaining the NZO and NZL strains that the males are susceptible to urogenital tract infections, sperm plugs, and pyelonephritis. We avoided these complications in the present study by supplementing drinking water with an antibiotic and maintaining breeding stock and aging males in pressurized, individually ventilated cages. Under these conditions, a group of 19 NZL-*Igh-6$^{null}$* males were produced for aging to 20 weeks without age-associated loss in body weight. With the larger group size, we failed to confirm the preliminary observation that B-lymphocyte deficiency significantly protected against eventual development of diabetes. A significant retardation of plasma glucose rise was observed at the 8-week sampling. Although mean plasma glucose level was also lower at the 12- and 16-week intervals, overall diabetic frequencies attained by 20 weeks were not significantly different between genotype (NZL 93%, NZL-*Igh-6$^{null}$* 79%). This result is consistent with an earlier finding in another mouse diabesity model, the C57BLKS/J-Lepr$^{db}$ mouse. Numerous studies had documented immune anomalies associated with this strain background susceptibility to diabesity; however, elimination of T- and B-lymphocyte components by genetic means only retarded severity without preventing establishment of chronic hyperglycemia [16]. Similarly, in the current study, B-lymphocyte immunodeficiency only retarded, but failed to prevent, the ultimate development of diabesity in NZL-*Igh-6$^{null}$* males maintained in PIV caging with antibiotic supplement to prevent infections. Hence, we conclude that our initial finding of suppressed diabesity [7] likely reflected the compromised health status (including severe pyelonephritis) of the initial small group of males in the absence of special procedures to protect against infections. Interestingly, diabetes development in NZL males achieves the same high frequency as the (NZO × NON)F1 hybrid male [17]. This increased penetrance of diabesity, coupled with the improved reproductive performance despite comparable obesity, makes the new NZL strain (without the *Igh-6$^{null}$* mutation) an attractive substitute for the NZO/HlLt strain.

The presence of low affinity IgM AIRAs in the sera of male NZO mice has been reported [6]. Using the mIR-transfected CHO cell system (mIR36.11.1) and flow cytometry, the presence of AIRAs in sera from young male NZO/HlLt was confirmed. In sera from 16- to 17-week-old NZO/HlLt males, when the diabetes incidence is about 10%, AIRAs were present in the IgM-enriched 25% SAS precipitate, but not in the IgG-enriched protein A eluate. This indicates that the initial appearance of AIRAs in NZO/HlLt males is primarily in the form of IgM. The presence of IgG AIRA activity in the sera of diabetic NZO/HlLt males by flow cytometry and radioimmunoassay, and its absence in mature normoglycemic NZO/HlLt males, appeared to correlate AIRA activity with the development of type 2 diabetes. In humans, AIRAs are associated with a rare type B syndrome of extreme insulin resistance. AIRAs block insulin binding and mimic the biological effects of insulin leading to insulin resistance and receptor desensitization [18, 19]. Recently, it was shown in a human patient that AIRAs produced insulin resistance by desensitizing signaling through the IR via inducing a stable association of the IR with IR substrates 1 and 2 [20]. In summary, comparison of NZL/Lt males with and without the ability to produce AIRAs has established that although these autoantibodies may be useful as markers of the disease state, AIRAs are not essential for development of type 2 diabetes in this model.

### REFERENCES

[1] Bielchowsky, M., and Bielschowsky, F. (1956) The New Zealand strain of obese mice. Their response to stilbestrol and to insulin. *Aust. J. Exp. Biol.,* **34,** 77–97.

[2] Veroni, M. C., Proietto, J., and Larkins, R. G. (1991) Evolution of insulin resistance in New Zealand obese mice. *Diabetes,* **40,** 1480–1487.

[3] Leiter, E. H., Reifsnyder, P. C., Flurkey, K., Partke, H-J., Junger, E., and Herberg, L. (1998) Non-insulin dependent diabetes genes in mice: Deleterious synergism by both parental genomes contributes to diabetogenic thresholds. *Diabetes,* **47,** 1287–1295.

[4] Melez, K. A., Attallah, A. M., Harrison, E. T., and Raveche, E. S. (1985) Immune abnormalities in the diabetic New Zealand obese (NZO) mouse: Insulin treatment partially suppresses splenic hyperactivity measured by flow cytometry analysis. *Clin. Immunol. Immunopathol.,* **36,** 110–119.

[5] Melez, K. A., Harrison, L. C., Gilliam, J. N., and Steinberg, A. D. (1980) Diabetes is associated with autoimmunity in the New Zealand obese (NZO) mouse. *Diabetes,* **29,** 835–840.

[6] Harrison, L. C., and Itin, A. (1979) A possible mechanism for insulin resistance and hyperglycemia in NZO mice. *Nature,* **279,** 334–336.

[7] Haskell, B. D., Flurkey, K., Duffy, T. M., Sargent, E. E., and Leiter, E. H. (2002) The diabetes-prone NZO/H1 strain. I. Immunophenotypic comparison to the related NZB/BinJ and NZW/LacJ strains. *Lab. Invest.,* **82,** 833–842.

[8] Junger, E., Herberg, L., Jeruschke, K., and Leiter, E. H. (2002) The diabetes-prone NZO/H1 strain: II. Pancreatic immunopathology. *Lab. Invest.,* **82,** 843–853.

[9] Kitamura, D., Roes, J., Kuhn, R., and Rajewsky, K. (1991) A B cell-deficient mouse by targeted disruption of the membrane exon of the immunoglobulin mu chain gene. *Nature,* **350,** 423–426.

[10] Taguchi, N., Hashimoto, Y., Hsu, T., Ansari, A. A., Shultz, L., Dorshkind, K., Ikehara, S., Naiki, M., and Gershwin, M. E. (2001) B cells are selectively associated with thymic cortical but not medullary pathology in NZB mice. *J. Autoimmun.,* **16,** 393–400.

[11] Swick, A. G., Janicot, M., Cheneval-Kastelic, T., McLenithan, J. C., and Lane, M. D. (1992) Promoter-cDNA-directed heterologous protein expression in *Xenopus laevis* oocytes. *Proc. Natl. Acad. Sci. U. S. A.,* **84,** 1812–1816.

[12] Zapf, A., Hsu, D., and Olefsky, J. M. (1994) Comparison of the intracellular itineraries of insulin-like growth factor-I and insulin and their receptors in Rat-1 fibroblasts. *Endocrinology,* **134,** 3445–3452.

[13] Formisano, P., Najjar, S. M., Gross, C. N., Philippe, N., Oriente, F., Kern-Buell, C. L., Accili, D., and Gorden, P. (1995) Receptor mediated internalization of insulin. Potential role of pp120/HA4, a substrate of the insulin receptor kinase. *J. Biol. Chem.,* **270,** 24073–24077.

[14] Whittaker, J., Okamoto, A. K., Thys, R., Bell, G. I., Steiner, D. F., and Hofmann, C. A. (1987) High level expression of human insulin receptor cDNA in mouse NIH 3T3 cells. *Proc. Natl. Acad. Sci. U. S. A.,* **84,** 5237–5241.

[15] Reifsnyder, P. C., and Leiter, E. H. (2002) Deconstructing and reconstructing obesity-induced diabetes (diabesity) in mice. *Diabetes.,* **51,** 825–832.

[16] Leiter, E. H., Prochazka, M., and Shultz, L. D. (1987) Effect of immunodeficiency on diabetogenesis in genetically diabetic (db/db) mice. *J. Immunol.,* **138,** 3224–3229.

[17] Leiter, E. H., Reifsnyder, P. C., Flurkey, K., Partke, H. J., Junger, E., and Herberg, L. (1998) NIDDM genes in mice: Deleterious synergism by both parental genomes contributes to diabetogenic thresholds. *Diabetes,* **47,** 1287–1295.

[18] Van Obberghen, E., Grunfeld, C., Harrison, L. C., Karlsson, A., Muggeo, M., and Kahn, C. R. (1982) Autoantibodies to the insulin receptor and insulin-resistant diabetes. *Horm. Res.,* **16,** 280–288.

[19] Kahn, C. R., Kasuga, M., King, G. L., and Grunfeld, C. (1982) Autoantibodies to insulin receptors in man: Immunological determinants and mechanisms of action. *Ciba Found. Symp.,* **90,** 91–113.

[20] Auclair, M., Vigouroux, C., Desbois-Mouthon, C., Deibener, J., Kaminski, P., Lascols, O.,Cherqui, G., Capeau, J., and Caron, M. (1999) Antiinsulin receptor antibodies induce insulin receptors to constitutively associate with insulin receptor substrate 1 and 2 and cause severe cell resistance to both insulin and insulin-like growth factor 1. *J. Clin. Endocrinol. Metab.,* **84,** 3197–3206.

